# Can Thailand achieve COVID‐19 herd immunity?

**DOI:** 10.1002/puh2.7

**Published:** 2022-06-07

**Authors:** Manatee Jitanan, Tharisara Chirasatienpon, Rapeeporn Tiamjan, Kwanjai Amnatsatsue, Rachanon Nguanjairak, Adriana Viola Miranda, Xu Lin, Dawa Gyeltshen, Paolo Miguel Manalang Vicerra, M. B. N. Kouwenhoven

**Affiliations:** ^1^ Department of Physical Education Faculty of Education Kasetsart University Bangkok Thailand; ^2^ Department of Public Health Faculty of Science and Technology Chiang Mai Rajabhat University Thailand; ^3^ Faculty of Public Health Mahidol University Bangkok Thailand; ^4^ Department of Community Public Health Faculty of Public Health Nakhon Ratchasima Rajabhat University Nakhon Ratchasima Thailand; ^5^ Faculty of Medicine Universitas Indonesia Jakarta Indonesia; ^6^ Department of Thoracic Surgery The First Affiliated Hospital School of Medicine Zhejiang University Hangzhou Zhejiang China; ^7^ Department of Internal Medicine Jigme Dorji Wangchuck National Referral Hospital Thimphu Bhutan; ^8^ Asian Demographic Research Institute Shanghai University Shanghai China; ^9^ Department of Physics Xi'an Jiaotong‐Liverpool University Suzhou China

**Keywords:** COVID‐19, herd immunity, Thailand

## Abstract

The COVID‐19 outbreak has had a great impact on the social, economic, and health systems of Thailand. A variety of measures to curb the spread of the disease were implemented since the beginning of the pandemic, including a strict national lockdown protocol. The Thai government aimed to achieve herd immunity through an efficient vaccination programme. Initially, vaccine supply shortage and a lack of vaccine options plagued the health system, but this has since been improved. Continuous monitoring of the situation through research is being carried out to assess the level of immunity among the population whereby the current general recommendation is presently a fourth booster dose for adults. Hurdles towards achieving herd immunity remain. One such issue is the low level of vaccine literacy among those that are unvaccinated or inadequately vaccinated. Another obstacle is the sizeable rate of hesitancy towards getting booster doses. Achieving herd immunity in the Thai population would require multilateral cooperation, improved health promotion to target population groups, such as older adults, and a developed distribution system for those with limited access, such as those in the rural areas.

## COVID‐19 PANDEMIC IN THAILAND

The first case of COVID‐19 in Thailand was reported in January 2020, and the first COVID‐19 surge started in March of the same year [1]. Public health measures, including a strict national lockdown policy, led to the decrease in the number of cases by May 2020 and the measures were gradually lifted. There was a resurgence of the number of cases between December 2020 and February 2021 [1]. This trend of resurgence happened in the background differing levels of adherence of the population to the public health measures, the lack of vaccines at that time and the newly emerging virus strains [1].

The infection rate in the country again started to increase rapidly in the second quarter of 2021, while the vaccination coverage of the eligible population remained low [2]. This wave started to slow down by December 2021 [3]. Vaccination rollout became vigorous with the emergence of the Omicron variant of COVID‐19 which had posed a threat to the social and economic situation of the country once again. During this time, studies were performed to provide focus on breakthrough infections and the need for a third vaccine dose [4]. This guided the government to implement such an action while continuing to emphasize the need for basic COVID‐19 related hygiene procedures for individuals and for business establishments.

The pandemic impacted many aspects of the society, particularly its economy and daily life. The tourism sector experienced a major blow because of the decreased number of flights globally, the strict regulations for flying, and the strict regulations for entering and exiting countries, and as well as the compulsory closure of tourism facilities. Lockdowns contributed to the inability of students to attend face‐to‐face classes, and for many citizens to lose their livelihoods [1].

## COVID‐19 VACCINE ROLLOUT IN THAILAND

The population of Thailand in 2021 was around 69.9 million individuals, where 64% were at the ages of 15–59 years, and 19% were at least 60 years old [5]. The country has a universal healthcare system and provides for all residents without charge [6]. A budget of 6 billion Baht (∼174 million USD) was announced for vaccine purchase in 2020; and for 2021, it was even stated that the budgetary allocation was unlimited [7]. By 21 April 2022, over 70% of the population has already been vaccinated with the primary series for COVID‐19 protection, and 36% has received at least one booster dose [3].

Currently, six vaccine manufacturers have been approved for emergency use in Thailand including Moderna, Pfizer, Johnson & Johnson, AstraZeneca, Sinopharm, and Sinovac [13]. During the earlier phase of the vaccine rollout, from February to May 2021, only the Sinovac vaccine was available [14]. Two million doses of said vaccine brand were administered to prioritized groups, including healthcare workers, citizens aged 60 years and older, and highly vulnerable adults with pre‐existing conditions. In June 2021, the AstraZeneca vaccine was also distributed in the country. In the fourth quarter of the same year, the recommendation for adults to receive the third dose of the vaccine was implemented. As time passed, the other vaccine brands became available, leading to the target procurement and deployment plan of the government of 100 million doses before 2022 [14].

## HERD IMMUNITY IN THAILAND

Herd immunity is realized when the population gains immunity through vaccination and/or previous exposure to infection [9]. The conditions for achieving herd immunity depend on the prevailing reproduction number (R_0_) of COVID‐19 in the population, and on the fraction of the infected population that is able to recover and gain immunity from the disease [15]. As there are two means of achieving herd immunity, it is important to reiterate that natural infection has associated risks, especially for health‐vulnerable individuals, and it also results in people remaining prone to reinfection [16]. Relevant literature has shown that the currently available vaccines offer significant protection against severe effects of the disease. Despite this, further studies should be carried out to determine the longevity of the immunity, as the virus continues to evolve [16, 17].

Thailand's commitment to achieve herd immunity is reflected in the "Integrated Plan for Multilateral Cooperation for Safety and Mitigation of Covid‐19" [18]. This plan aims to reduce the chance of the virus transmission, mitigate the health, economic, and social impacts of the pandemic, and increase national security. The government has regularly provided daily updates on COVID‐19 pandemic through a number of media avenues. These included information about new restrictions, safety precautions, and official news concerning related to the pandemic. Societal participation and cooperation played an important role in governance and decision making for pandemic responses.

In addition, the Ministry of Public Health released a plan of action in August 2021 that discussed ways to prevent and control COVID‐19 at home and in specific public settings, such as business establishments. Standards were set for businesses to be able to operate, and these measures were dubbed as the *Bubble and Seal* method [19]. Two components were part of the said method—(1) basic health protocols, such as wearing of masks, regularly monitoring body temperatures, and ensuring that at least 70% of the employees were vaccinated, and (2) isolation if positive cases were detected among employees.

Amongst these social measures, the vaccination drive is at the centre of the abovementioned plan of the Ministry of Public Health. Initially, vaccine supply was limited; therefore, the Ministry of Public Health established allocation guidelines to maintain the viability of the health service system [20]. The first phase of vaccine delivery was intended for medical workers, public health personnel, front‐line workers, persons with selected comorbidities, and persons aged 60 years and older. After a sufficient proportion of people belonging to these groups was vaccinated, the rest of the population were to be covered. Following the development of the pandemic and the progress of the vaccination program, supply allocation shifted. The county's priority group eventually included those who were viewed as economically‐viable, particularly people in the tourism industry. The target group included people involved in local tourism activities and those who required international travel, such as the crews of airplanes and ships. Workmen were also prioritized, with the intend to move the economy back towards normalization.

The report of the country's Department of Disease Control on 21 April, 2022 stated that about 81% of the population received at least one dose of the vaccine; 73% received the complete primary series; and 36% were given a third dose [3]. It is also important to highlight the geographic distribution of the vaccine coverage. The entire population of Bangkok, the capital city, received at least one dose. The nearby provinces also have high rates of people who received first dose, including and Chonburi. Areas that are more distant from Bangkok have a notably lower vaccine coverage.

To reflect the vaccination progress and the current pandemic situation, Thailand has recorded more than 15,000 positive cases each day since February 2022 [3]. This rate is comparable to the months in 2021 when lockdown measures were implemented [21]. The difference between the periods is that the rates of severe cases and hospital occupancy, in general, have been much lower in recent months. However, vigilance is still exercised by monitoring risk groups in the provinces, as they have lower vaccination rates than Bangkok and its peripheral areas.

## OBSTACLES FOR HERD IMMUNITY

While the government had emphasized the need for an efficient vaccine rollout, paucity in vaccine options and supply led to public outcry early in the pandemic [8, 9]. At the onset of the global vaccine rollout, Thailand's vaccine supply was not secured, such that the first vaccination was done on 28 February 2021. This is late compared to neighbouring countries like Laos, which had its first vaccination by November 2020 [10]. Among the factors that affected the supply inadequacy was Thailand's rejection of support from the COVAX programme, the COVID‐19 Vaccines Global Access Facility. According to the Thai government, it was because the country was a middle‐income nation that could not receive vaccines for free or for a low price through this program [12]. Although its government opted out from accessing the COVAX facility, it received donated vaccines from other countries to augment its supply at the time. Following the public outcry, the government also enhanced its vaccine procurement, indicating that at least two brands of vaccines would be available at the end of 2021 [2, 11].

In addition to supply limitations at the time, the vaccine rollout was also hampered by the country's inefficient vaccine allocation model. An epicentre‐based strategy was used until recently when lockdowns were being implemented which allocated the vaccines where prevalence of COVID‐positive cases were detected [11]. The epidemiology of COVID‐19 was ignored, notably the aspects of demographic distribution and the dynamics of transmissibility itself.

Low vaccine literacy and the resulting vaccine hesitancy posed additional challenges for the Thai society's vaccination program. It was observed that about 80% of the sample in Chiang Mai demonstrated sufficient vaccination knowledge in May 2021 [22], the vaccine acceptance rate was only at around 42% among local residents [2]. Information on the efficacy of vaccine types, as well as their respective mild and adverse effects were known to only few, primarily those in the medical field [8]. The general population was wary because much of the information, particularly found on social media, was overwhelming. The self‐perceived level of knowledge about efficacy level and side effects, among other aspects, was high. However, there was low discernment on which aspect of such information was true and reliable. At the same time, the differing efficacy levels of the different vaccines also fuelled vaccine hesitancy in the country. A 2021 survey indicated that the vaccine acceptance rate would improve by up to 89.0–91.3% if the residents would be allowed to select the vaccines for themselves—a condition that has a low cost‐benefit ratio considering the limited vaccine supply and the relatively high protection that even vaccines with lowest efficacy level provide, when compared to no vaccination at all.

Hesitancy among the identified vulnerable populations has also been noted to be prevalent, especially among older people [23]. Adults aged at least 60 years were identified as among the top prioritised group in relation to the COVID‐19 response. Certain issues though remain unresolved such that about 44% of participants in a small‐sample study carried out in the third quarter of 2021 were vaccine‐hesitant. A confluence of background characteristics enforced such reluctance including, but not limited to, vaccine manufacturer options, a low level of education, and a general lack of confidence in the health system.

Aside from the issues on the part of the public and their knowledge and acceptance of vaccinations, the vaccines themselves may also pose challenges to achieving herd immunity. The efficacy of COVID‐19 vaccines wane over time, which has led to the decision to introduce booster doses in the country [4, 24]. A number of studies were carried out [4, 24] and are currently being carried out [25] to track antibody levels among the population vis‐à‐vis the development of SARS‐CoV‐2. Thailand's National Vaccine Institute has favoured a fourth dose, especially for older people and those with comorbidities, since the advent of the Omicron and its evolving variants [26]. The said institute cited that a third dose prevents mortality by 98% and a fourth dose can improve the said risk even further. Despite costs and actions required to introduce multiplicity of vaccinations, it is recognised that it is the optimal method for a population to be protected through immunisation, rather than through the experience of natural infection [16].

## CONCLUSION AND RECOMMENDATIONS

Attaining herd immunity is a challenge in Thailand because of a number of obstacles. The restrictions on vaccine choice, inadequate vaccine literacy, low acceptance rate of vaccination, and high vaccine hesitancy among seniors are among the challenges that Thailand needs to address to achieve herd immunity. Individuals need to be convinced to receive the recommended booster doses, preferably until the fourth dose as recommended by the government, to continue building protection within communities. Observing the guidelines on practicing basic health protocols (e.g., wearing a mask) and getting vaccinated are anchored in trust on the health system. Increased communication transparency concerning the disease and the vaccines is crucial as people become better equipped with sufficient and reliable information.

To achieve herd immunity, the Thai government could work on having an efficient policy that covers many aspects of COVID‐19, constructing an integrated plan for multi‐sectoral cooperation for safety and mitigation of COVID‐19, formulating a scheme to provide vaccines to target groups, and building a strong system of prevention and control in specific geographical areas.

## CONFLICT OF INTEREST

The author declares no competing interests.

## FUNDING INFORMATION

There is no funding in the development for this paper.

## AUTHOR CONTRIBUTIONS

Manatee Jitanan: Conceptualization; Data curation; Formal analysis; Writing – original draft; Writing – review & editing. Tharisara Chirasatienpon: Data curation; Formal analysis; Writing – original draft. Rapeeporn Tiamjan: Data curation; Formal analysis; Writing – original draft. Kwanjai Amnatsatsue: Data curation; Formal analysis; Writing – original draft. Rachanon Nguanjairak: Data curation; Formal analysis; Writing – original draft. Xu Lin: Supervision; Writing – review & editing. Paolo Miguel Manalang Vicerra: Supervision; Writing – review & editing

## Data Availability

No database or primary data was used in preparing the manuscript.
